# Local Interleukin-12 Treatment Enhances the Efficacy of Radiation Therapy by Overcoming Radiation-Induced Immune Suppression

**DOI:** 10.3390/ijms221810053

**Published:** 2021-09-17

**Authors:** Ching-Fang Yu, Chun-Hsiang Chang, Chun-Chieh Wang, Ji-Hong Hong, Chi-Shiun Chiang, Fang-Hsin Chen

**Affiliations:** 1 Radiation Biology Research Center, Institute for Radiological Research, Chang Gung Memorial Hospital Linkou Branch, Chang Gung University, Taoyuan 33302, Taiwan; chingfang@mail.cgu.edu.tw (C.-F.Y.); jjwang@cgmh.org.tw (C.-C.W.); jihong@cgmh.org.tw (J.-H.H.); 2 Department of Radiation Oncology, Chang Gung Memorial Hospital Linkou Branch, Taoyuan 33382, Taiwan; 3 Department of Biomedical Engineering and Environmental Sciences, National Tsing Hua University, Hsinchu 30013, Taiwan; doo1002000@yahoo.com.tw; 4 Department of Medical Imaging and Radiological Sciences, Chang Gung University, Taoyuan 33302, Taiwan; 5 Institute of Nuclear Engineering and Science, National Tsing Hua University, Hsinchu 30013, Taiwan; 6 Frontier Research Center on Fundamental and Applied Sciences of Matters, National Tsing Hua University, Hsinchu 30013, Taiwan

**Keywords:** radiation, prostate cancer, interleukin-12, T cell exhaustion, vascular maturation, liver toxicity

## Abstract

Radiation therapy (RT) recruits myeloid cells, leading to an immunosuppressive microenvironment that impedes its efficacy against tumors. Combination of immunotherapy with RT is a potential approach to reversing the immunosuppressive condition and enhancing tumor control after RT. This study aimed to assess the effects of local interleukin-12 (IL-12) therapy on improving the efficacy of RT in a murine prostate cancer model. Combined treatment effectively shrunk the radioresistant tumors by inducing a T helper-1 immune response and influx of CD8+ T cells. It also delayed the radiation-induced vascular damage accompanied by increased α-smooth muscle actin-positive pericyte coverage and blood perfusion. Moreover, RT significantly reduced the IL-12-induced levels of alanine aminotransferase in blood. However, it did not further improve the IL-12-induced anti-tumor effect on distant tumors. Upregulated expression of T-cell exhaustion-associated genes was found in tumors treated with IL-12 only and combined treatment, suggesting that T-cell exhaustion is potentially correlated with tumor relapse in combined treatment. In conclusion, this study illustrated that combination of radiation and local IL-12 therapy enhanced the host immune response and promoted vascular maturation and function. Furthermore, combination treatment was associated with less systemic toxicity than IL-12 alone, providing a potential option for tumor therapy in clinical settings.

## 1. Introduction

Prostate cancer is the most common cancer to be diagnosed among men in the USA [1]. Radical prostatectomy and radiation therapy are the standard primary treatments for patients with early-stage prostate cancer, while androgen-deprivation therapy is the main therapy for recurrent disease and/or advanced-stage prostate cancer. Despite the advance in therapeutic treatment, prostate cancer remains the second leading cause of cancer-related death in American men because most patients progress to metastatic castration-resistant prostate cancer [2,3]. Therefore, the development of effective therapy is still the main subject for prostate cancer therapy. Prostate cancer presents an immunosuppressive microenvironment, including lack of CD8+ lymphocytes’ infiltration, downregulation of major histocompatibility complex (MHC) expression, and higher expression of programmed cell death-ligand 1 (PD-L1) [4,5,6]. Therefore, turning from “cold” tumors into “hot” tumors may be a potential strategy to enhance immune response of prostate cancer.

In addition to surgery and systemic chemotherapy, radiation therapy (RT) is a frequently used and important treatment for numerous types of cancer. It can significantly prolong the survival time of patients and provide local tumor control. The cytotoxic mechanism of RT involves the direct killing of tumor cells, as well as the induction of systemic, immune-mediated tumor regression. The latter is achieved by increasing tumor antigen release and the cross-presentation of antigen-presenting cells, activating dendritic cells and enhancing T-cell priming. However, RT also stimulates chemokine signals, which recruit myeloid cells such as tumor-associated macrophages (TAMs) and myeloid-derived suppressor cells, causing suppression of T-cell immunity [7,8,9]. These factors may impede the anti-tumor efficacy of RT. Combining RT with other treatments to block or reverse the immunosuppressive effects has been considered to be a potential strategy for improving the effects of conventional therapies.

Owing to its important role in the activation of innate and adaptive immunity, interleukin-12 (IL-12) is an ideal candidate for cancer therapy. IL-12 is produced by activating antigen-presenting cells, such as macrophages, dendritic cells, and B cells [10]. The pivotal actions of IL-12 are to induce interferon-gamma (IFN-γ) production by natural killer and T cells [11,12,13,14], differentiate naïve CD4+ T cells toward the T helper-1 (Th1) phenotype [15], expand the growth and cytotoxic activity of natural killer and T cells [16], and enhance the antibody-dependent cellular cytotoxicity against tumor cells [17]. IL-12 also modifies the tumor microenvironment via enhancement of MHC class I expression on tumor cells [18,19], polarization of M2 macrophages toward activated M1 macrophages [20], and introduction of an anti-angiogenic effect by inhibiting the proliferation of endothelial cells associated with monokines induced by IFN-γ (MIG) and IFN-γ-inducible protein 10 (IP-10) [21,22]. Based on its ability to elicit the innate and adaptive immune responses and an anti-angiogenic effect, IL-12 has been regarded as a potential treatment option for the eradication of tumors. IL-12 monotherapy showed high therapeutic efficacy in various preclinical animal models [23,24]. The anti-tumor activity of IL-12 could be further improved by using IL-12 in combination with other strategies, such as chemotherapy, radiotherapy, and cytokines [25,26,27]. However, the occurrence of severe side effects poses a challenge to the clinical application of IL-12 therapy [28,29]. Strategies aiming to reduce the IL-12-induced toxicity could be employed to overcome this obstacle in the clinical setting. 

Our previous studies showed that high-dose RT temporarily retarded the growth of a radio-resistant prostate tumor and attracted the infiltration of TAMs with high levels of arginase-I (Arg-I), inducible nitric oxide synthase (iNOS), and cyclooxygenase-2 (COX-2) expression. This effect resulted in an immunosuppressive tumor microenvironment and promoted tumor regrowth [30]. RT led to the development of chronic hypoxic regions by decreasing the microvascular density (MVD) of tumors. M2 macrophages [31] and myeloid-derived suppressor cells [32] are attracted to and aggregate in chronic hypoxic and necrotic areas, respectively. These results demonstrated that the irradiated TRAMP-C1 prostate tumors presented an immunocompromised microenvironment. The aim of this study was to overcome this immunosuppression and enhance anti-tumor activity against radio-resistant prostate cancer by combining IL-12 immunotherapy with RT.

## 2. Results

### 2.1. IL-12 Therapy Enhanced the Anti-Tumor Activity of RT

**Figure 1:**
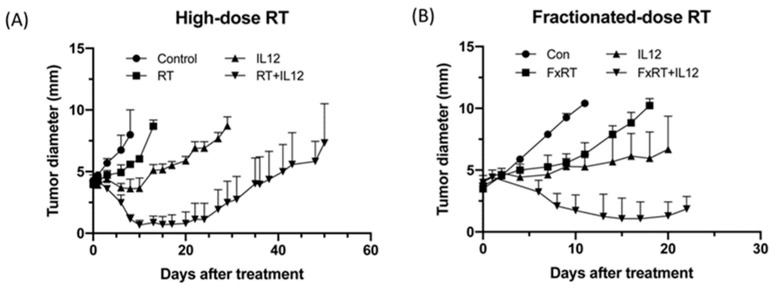
Combining radiation with local IL-12 therapy shrank TRAMP-C1 tumors and retarded the tumor growth. TRAMP-C1 tumors were irradiated when tumor reached the suitable size. Normal saline or Ad-sc-IL-12 adenovirus diluted in normal saline was injected after irradiation with (**A**) high-dose RT (25 Gy) and (**B**) fractionated-dose RT (8.9 Gy per fraction with five times). Each group contained four mice and the experiment was repeated twice independently.

Our previous study showed that high-dose RT (25 Gy) delayed the growth of TRAMP-C1 prostate tumors by approximately 1 week compared with control tumors [30]. However, it did not lead to tumor shrinkage. These findings indicated that TRAMP-C1 tumors were resistant to radiotherapy. Furthermore, TAMs of irradiated TRAMP-C1 tumors expressed a more immunosuppressive phenotype and exhibited higher tumor-promoting ability, which may also explain the resistance of TRAMP-C1 tumors to RT. The present study examined whether IL-12 immunotherapy could stimulate the immune response to enhance the efficacy of RT against radio-resistant TRAMP-C1 tumors. Tumor growth measurement showed that RT or IL-12 alone retarded TRAMP-C1 tumor growth by 7 and 12 days, respectively. Combined modality treatment effectively shrank TRAMP-C1 tumors within 20 days (tumor size < 1 mm) and extended the delay in tumor growth up to 40 days (Figure 1A). Remarkable anti-tumor results were also observed with the fractionated-dose RT regimen (five times of 8.9 Gy per fraction) with a similar biological effective dose to that of a single high-dose RT (25 Gy). Combination of fractionated-dose RT and IL-12, where Ad-sc-IL12 virus particles were injected after the first fraction, also significantly reduced the tumor size (Figure 1B). These data demonstrated that IL-12 immunotherapy enhanced the anti-tumor activity of RT, regardless of combination with high- or fractionated-dose RT, and significantly prolonged the delay in tumor growth.

### 2.2. Combined RT and IL-12 Therapy Induced Systemic Anti-Tumor Activity

**Figure 2:**
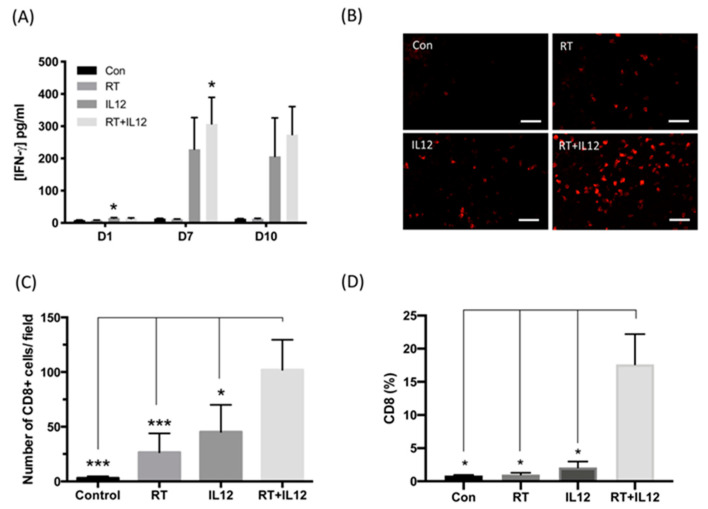
The anti-tumor immunity was strongly activated in combined modality treatment. (**A**) The level of IFN-γ in plasma at days 1, 7, and 10 post treatment were examined by ELISA assay. *n* = 3 in each group at day 1 and *n* = 4 in each group at days 7 and 10. (**B**) The representative figure of CD8+ T cells in tumor section of each group. Scale bar: 50 μm. (**C**) The number of CD8+ T cells was quantified by counting the positive signal under 400× magnification field in each section. Each section had at least 10 fields for quantification. Each group had at least three samples. (**D**) The population of CD8+ T cells in tumor cell suspension was analyzed by flow cytometry. *n* = 4 in control, RT, and IL-12 groups. *n* = 7 in RT+IL-12 group. * *p* < 0.05, *** *p* < 0.001.

The level of IFN-γ production in blood was examined on the indicated days after treatment, as an index of Th1 response, to assess the promotive effect of IL-12 on the activity of RT against TRAMP-C1 tumors. Following monotherapy with RT, the amount of IFN-γ in blood was low at all time points and not significantly different compared with that measured in the control group. Nonetheless, the levels of IFN-γ in blood were slightly increased at day 1 after the administration of the Ad-sc-IL12 virus and peaked at day 7, regardless of treatment regimen: IL-12 alone (228.7 ± 98.04 vs. con: 13.51 ± 0.85 pg/mL, *p* = 0.0703) or combined modality therapy (307.3 ± 82.06 vs. con: 13.51 ± 0.85 pg/mL, *p* = 0.0135). Sustained IFN-γ levels were found until day 10 after treatment: IL-12 alone (206.5 ± 119.2 vs. con: 12.97 ± 0.72 pg/mL, *p* = 0.1965) and combined modality therapy (273.8 ± 86.73 vs. con: 12.97 ± 0.72 pg/mL, *p* = 0.0556) (Figure 2A). These results indicated that the IL-12-induced immune response was not compromised by RT. 

To further investigate the effect of combined modality treatment on TRAMP-C1 tumors, the number of cytotoxic CD8+ T cells in tumors was assessed by immunohistochemistry staining in each group at day 7 after treatment. In control TRAMP-C1 tumors, CD8+ cells were rarely found (3.030 ± 0.9853 cells per field) within tumors, indicating that TRAMP-C1 is an intrinsic T-cell-deficient tumor (Figure 2B,C). Either RT or IL-12 alone induced the infiltration of few CD8+ T cells into local tumor sites (RT: 26.54 ± 7.771 cells per field; IL-12: 45.3 ± 14.30 cells per field); nevertheless, the number of cells was not significantly different compared with that observed in the control group. However, the combined modality therapy triggered extensive infiltration of CD8+ T cells into the tumors (102.0 ± 12.31 cells per field) versus control or monotherapy. Quantitative analysis of CD8+ T cells was further performed using flow cytometry. The percentage of CD8+ cells showed an increase in the group treated with IL-12 and marked elevation in the combined modality therapy group (Figure 2D). These results demonstrated that the combined modality therapy exerted a stronger Th1 immune response and recruited more CD8+ T cells into T cell-deficient TRAMP-C1 tumors compared with monotherapy.

### 2.3. Combined Modality Therapy Promoted Vascular Maturation

**Figure 3:**
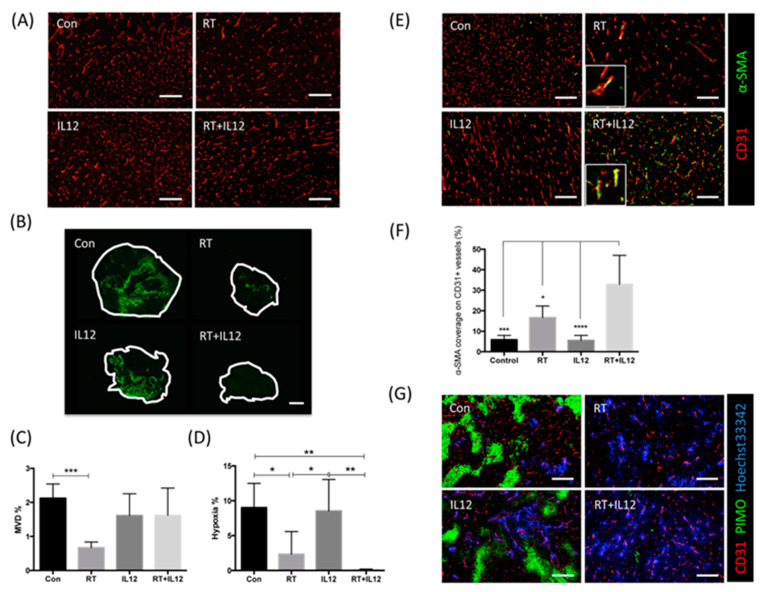
Combined modality therapy promoted the vascular maturation and function. Representative images of tumors stained by (**A**) CD31 and (**B**) PIMO to identify vessels and tumor hypoxia area. Scale bar: 200 μm and 1 mm in 3A and 3B, respectively. (**C**,**D**) The quantification of microvascular density (MVD) and hypoxia region was analyzed from the section of whole tumor area in each group. Each group had at least four samples. (**E**) Representative images of the coverage of CD31+ vessels (red) by α-SMA+ pericyte (green). Scale bar: 200 μm. (**F**) The percentage of pericyte coverage was examined under 100x magnification field in each section. Each sample had at least five fields for quantification. Each group had at least four samples. (**G**) Representative images of vessel (red), hypoxia (green), and blood perfusion (blue) in tumors. The blood perfusion was presented by Hoechst33342 signal. Scale bar: 200 μm. * *p* < 0.05, ** *p* < 0.01, *** *p* < 0.001, **** *p* < 0.0001.

IL-12 therapy exerts anti-tumor immunity and has anti-angiogenic ability. To investigate the anti-angiogenic role of IL-12 in TRAMP-C1 tumors, the microvascular density (MVD) of tumors at day 7 after treatment was quantified by staining with endothelial cell marker CD31 (Figure 3A). Based on our previous report, RT significantly decreased the MVD in TRAMP-C1 tumors compared with control tumors. In contrast, IL-12 alone led to a slight and nonsignificant decrease in vessel density (Figure 3A,C). More interestingly, the combined modality therapy did not effectively reduce the MVD, even in tumors irradiated with high-dose RT. To further assess the anti-angiogenic effect of the combined modality therapy, the relative MVD was examined on days 1, 7, and 10 post treatment (Supplementary Figure S1). High-dose RT decreased the relative MVD to 80%, 52%, and 45% at days 1, 7, and 10, respectively, after irradiation compared to the values obtained for the control group. IL-12 alone decreased the MVD until day 7 post treatment; however, the difference was not significant. In those that received combined modality therapy, the MVD did not change at day 1, slightly decreased at day 7, and significantly declined at day 10 post RT, similar to the RT group. This result suggested that IL-12 may delay the death of endothelial cells induced by RT. Furthermore, the combined modality therapy resulted in an extremely low hypoxic area in tumors versus other groups (Figure 3B,D). This implied that the vessels in tumors of mice that received combined modality therapy may have good perfusion function. To test this hypothesis, tumor vessels were examined using pericyte coverage and the pattern of Hoechst33342 dye perfusion for structural and functional analysis, respectively (Figure 3E,G). The integrity of tumor vessels was determined through the coverage of CD31+ endothelial cells by α-smooth muscle actin-positive (α-SMA+) pericytes (Figure 3E). Tumors from the control and IL-12 alone groups had low α-SMA+ expression and low levels of pericyte coverage (Figure 3F). RT increased the coverage of endothelial cells by α-SMA+ pericytes and improved the vascular perfusion (Figure 3F,G), suggesting vascular normalization after RT. More α-SMA+ cells were recruited into tumor sites of mice that received the combined modality therapy, and the level of coverage of CD31+ cells by α-SMA+ pericytes was significantly higher than those recorded in the other three groups (Figure 3F). These effects were accompanied by a well-perfused tumor microenvironment detected through Hoechst33342 dye perfusion (Figure 3G).

### 2.4. Combined Modality Therapy Reduced the Occurrence of Side Effects Associated with IL-12 in Liver Injury

**Figure 4:**
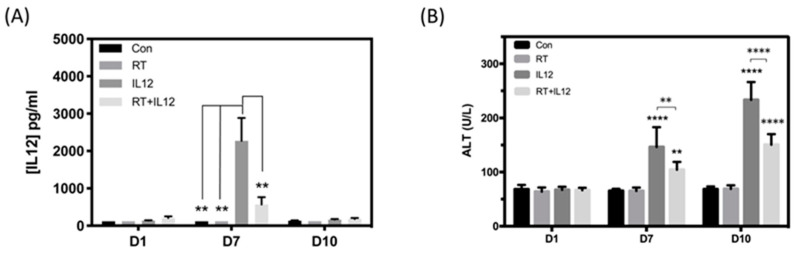
Combined modality therapy reduced the IL-12-induced toxicity on normal tissues. (**A**) The amount of IL-12 in plasma at days 1, 7, and 10 post treatment was quantified by ELISA assay. *n* = 3 in each group at day 1 and *n* ≥ 4 in each group at days 7 and 10. (**B**) The level of ALT in plasma was measured to assess the liver damage induced by IL-12 treatment. *n* ≥ 5 in each group. ** *p* < 0.01, **** *p* < 0.0001.

Previous studies indicated that systemic circulation of IL-12 is highly toxic to normal tissues, resulting in splenomegaly and liver toxicity; this has limited the application of IL-12 therapy in the clinical setting. Dynamic regulation of the systemic levels of IL-12 may be an effective approach to achieving better tumor control and preventing damage to normal tissues in clinical use. The levels of IL-12 in blood were monitored at the indicated days after infection with the virus. As shown in Figure 4A, IL-12 was slightly increased in the IL-12 alone group versus the control group at day 1 after infection (141.3 ± 8.137 vs. 118.3 ± 0.1157 pg/mL, respectively) and significantly elevated at day 7 post treatment (2272 ± 613.9 vs. 118 ± 0.8654 pg/mL, *p* = 0.0046, respectively). In contrast, tumors treated with the combined modality therapy exhibited markedly lower production of IL-12 at day 7 as compared with those in the IL-12 alone group (568.1 ± 197.1 vs. 2272 ± 613.9 pg/mL, *p* = 0.0098). The amounts of IL-12 in both groups returned to the basal levels at day 10 after treatment. 

Alanine aminotransferase (ALT) is an enzyme produced by cells in the liver and used as an indicator for liver damage or toxicity. The levels of ALT in the control and RT groups did not change during the experimental period (Figure 4B). In the IL-12 alone group, the levels of ALT were significantly increased at day 7 and showed marked elevation at day 10. However, the combined modality therapy group demonstrated significantly attenuated levels of ALT at both days 7 and 10. These results suggested that combined modality therapy significantly reduced the systemic toxicity associated with IL-12 by limiting the production of IL-12, thereby minimizing liver damage.

### 2.5. Radiation Therapy Did Not Enhance the Anti-Tumor Effect of IL-12 Immunotherapy on Distant Tumors

**Figure 5:**
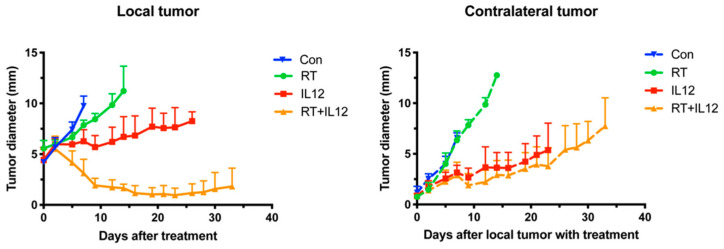
RT did not further improve IL-12-mediated anti-tumor effect on distant tumors. An equal number of TRAMP-C1 tumor cells were implanted in the right and left legs, respectively, of mice at 4-day intervals. The tumors of right legs were treated with irradiation and/or IL-12 therapy when tumor grew up about 4 mm. The growth of local and contralateral tumors was measured every 2 days with at least three mice in each time point.

It has been reported that RT induces spontaneous tumor regression outside the irradiation field in some cancers; this process is termed the “abscopal effect” [33]. The above data demonstrated that the combination of IL-12 therapy with RT induced a highly synergistic effect. We further investigated whether the combined modality therapy evoked systemic anti-tumor activity on distant tumors. For this purpose, mice were first inoculated with tumor cells in their right legs (local site) and received treatments when the local tumor size reached 4 mm. Four days after injection of tumor cells in the right legs, an equal number of tumor cells were inoculated into the left legs (contralateral site) and tumor growth was monitored. The results showed that contralateral tumors in the RT group continued to expand at the same growth rate as those of the control group, indicating that RT alone did not induce the abscopal effect (Figure 5). Intratumoral administration of IL-12 alone inhibited local tumor growth and significantly retarded the growth rate of contralateral tumors. The combined modality therapy effectively suppressed local tumor growth and exerted a similar therapeutic effect to that of IL-12 alone on contralateral tumors. These results revealed that IL-12 therapy significantly improved the efficacy of RT against local tumors. Moreover, the IL-12-induced anti-tumor effect on distant tumors was not compromised by RT.

### 2.6. Treatment with IL-12 Resulted in the Dysfunction of Tumor-Infiltrating T Cells

**Figure 6:**
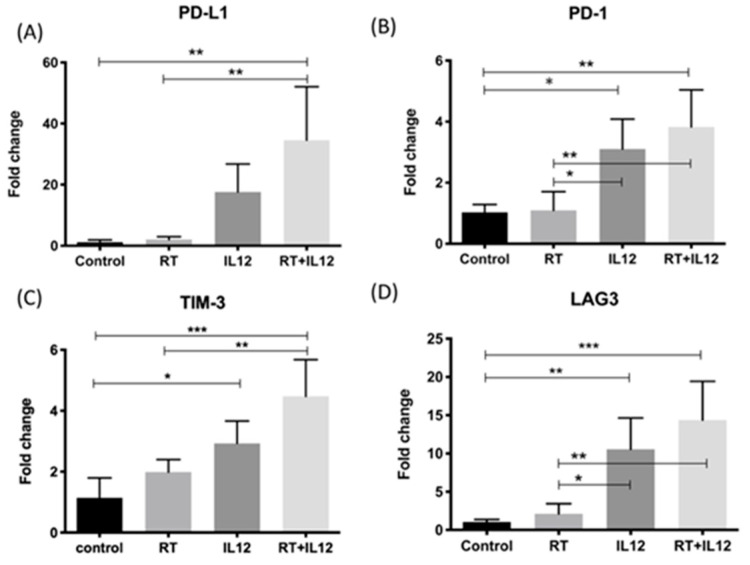
The T cell exhaustion-associated genes were upregulated in IL-12 alone and combined modality groups. The gene expression levels of (**A**) PD-L1, (**B**) PD-1, (**C**) TIM-3, and (**D**) LAG-3 in each tumor at day 7 after treatments was examined by real-time PCR. *n* = 4 in each group. * *p* < 0.05, ** *p* < 0.01, *** *p* < 0.001.

The combined modality therapy significantly repressed the growth of TRAMP-C1 tumors and enhanced anti-tumor activity by inducing a systemic Th1 immune response and recruiting more CD8+ T cells to the local tumor site. Nevertheless, mice that received combined modality therapy relapsed after day 20. To investigate whether the anti-tumor activity was attenuated during tumor progression, the number of CD8+ T cells was first assessed using immunohistochemistry staining. The number of CD8+ T cells in tumors of mice treated with the combined modality regimen declined at day 20 after treatment (12.08 ± 2.256 cells per field) and remained low (13.96 ± 2.741 cells per field) until tumors regrew to a size of 8 mm at the end stage of the experiments. Genes associated with T cell exhaustion in tumor tissues at day 7 post treatment were examined using quantitative PCR. The findings revealed that the expression of programmed cell death-ligand 1 (PD-L1), programmed cell death 1 (PD-1), T cell immunoglobulin and mucin domain 3 (TIM-3), and lymphocyte-activation gene 3 (LAG-3) was upregulated in tumors treated with IL-12 alone and reached markedly higher levels in those that received the combined modality therapy after day 7 (Figure 6). These observations indicated the process of T cell exhaustion. The results suggested that the combined modality therapy exhibited strong anti-tumor activity and induced negative regulatory pathways in tumors, especially concerning the modulation of cytotoxic T cell function. These effects may be linked to tumor relapse after combination treatment.

## 3. Discussion

The development of an immunosuppressive tumor microenvironment in malignant tumors that reduces the anti-tumor activity of RT compromises the curative effect of this treatment modality. Therefore, the reversal of this immunosuppressive tumor microenvironment may improve the outcomes of RT. This study showed that the combination of RT and local IL-12 therapy significantly shrank radio-resistant TRAMP-C1 tumors and retarded their growth by inducing a Th1 immune response and recruiting numerous cytotoxic CD8+ T cells into tumors. Additionally, the combined modality regimen delayed radiation-induced vascular death and improved vascular maturation by recruiting more α-SMA+ pericytes to cover endothelial cells. In addition, RT reduced the liver toxicity induced by treatment with IL-12 by efficiently killing virus-infected tumor cells through RT. However, it did not further enhance the anti-tumor effect induced by systemic IL-12 on distant TRAMP-C1 tumors. Interestingly, genes related to T cell exhaustion were simultaneously upregulated in tumors treated with IL-12. This upregulation may reduce the anti-tumor immunoactivity induced by the combined modality therapy.

IL-12 is a potent anti-angiogenic factor in tumors, potentially involving the activation of MIG and IP-10. The anti-angiogenic mediators MIG and IP-10 in tumors were upregulated at day 7 after treatment with IL-12 (Supplementary Figure S2). This process may have led to the slightly decreased vascular density observed in the TRAMP-C1 prostate tumor model (Figure 3). RT also exerts an anti-angiogenic effect on TRAMP-C1 prostate tumors. At a high dose (25 Gy), RT decreased the MVD to 80%, 52%, and 45% at days 1, 7, and 10 after RT, respectively (Supplementary Figure S1) [31]. However, the combination of RT with IL-12 did not show a synergistic anti-angiogenic effect. The MVD was almost unaltered at day 1, mildly declined at day 7, and decreased to a level similar to that noted in the RT group at day 10 post RT (Figure 3C and Supplementary Figure S1). This implied that the combined modality regimen induced an unexpected vascular modulation to mitigate the death of endothelial cells caused by radiation. Nevertheless, further research is warranted to elucidate the mechanism involved in this process. The combined modality therapy markedly increased the coverage of vessels by α-SMA+ pericytes and enhanced vascular perfusion. This suggested that, in the combined modality regimen, IL-12 immunotherapy activates systemic immunity and enhances the vascular normalization of irradiated tumors.

In clinical trials, the strategy for the administration of IL-12 recombinant protein involves high dosage and multiple deliveries due to its short half-life. However, the systemic administration of IL-12 is accompanied by some lethal syndromes (e.g., anemia, lymphopenia, neutropenia, muscle and hepatic toxicities, enlargement of lymph nodes, and splenomegaly) that limit the application of IL-12 [34,35]. Numerous studies have focused on improving the delivery strategies to avoid the development of systemic toxicity and enhance the anti-tumor efficacy of IL-12 therapy. The fusion protein NHS-IL12 consists of the functional domain of IL-12 and the antibody NHS76 that specifically binds to naked histone/DNA complexes in necrotic areas of tumors. When combined with IL-12, this protein improved the therapeutic efficacy and caused long-term tumor suppression [36]. Intravenous injection of NHS-IL12 did not cause acute or long-term systemic toxicity. NHS-IL12 combined with radiation-arrested tumor growth and significantly improved the survival [27]. Wang et al. designed a tumor-targeted oncolytic adenovirus (Ad-TD) to deliver non-secreting IL-12 (nsIL-12) to peritoneally disseminated and orthotopic pancreatic tumors [37]. Modified Ad-TD-nsIL-12 released IL-12 only when tumor cells were lysed after viral infection, thereby restricting the spread of IL-12 in the local tumor site. Systemic administration of Ad-TD-nsIL-12 did not induce a toxic effect on liver tissues of tumor-bearing mice. The present study showed that the strong anti-tumor activity of IL-12 was exerted following the initial infection of tumor cells with a virus carrying IL-12. High-dose irradiation caused tumor death (mitotic death), thereby easing the secretion of IL-12 into peripheral blood after tumor shrinkage. Our strategy demonstrated that the combination of RT and local IL-12 treatment could simultaneously evoke anti-tumor immunity and reduce toxicity. The combined modality therapy caused significant tumor shrinkage and delayed tumor regrowth by inducing a strong Th1 immune response. Treatment of mice with the combined modality therapy reduced the production of IL-12 by four-fold compared with that recorded after treatment with IL-12 alone at day 7. Moreover, the levels of ALT in the combined modality therapy group were significantly lower (1.5-fold) than those measured in the IL-12 alone group at day 10 after treatment (Figure 4). These results indicated that the combined modality regimen effectively reduced the systemic toxicity and elicited strong anti-tumor activity. This therapeutic strategy may provide a new option for the treatment of tumors in clinical settings.

The high anti-tumor efficacy of IL-12 has been demonstrated in numerous preclinical animal models and some clinic trials [23,24,38]. Combined radiation and IL-12 eradicated large orthotopic hepatocellular carcinoma by increasing antigen-presenting activity, reducing MDSCs’ accumulation, and activating CD8+ T and NK cells [39]. This study indicated that the combined modality therapy induced a strong immune reaction to suppress radioresistant TRAMP-C1 tumors. However, the combined modality therapy did not further improve the anti-tumor effect on distant sites induced by treatment with IL-12 alone. An influx of CD8+ T cells recruited into tumors and a significant elevation of IFN-γ in plasma were found in mice that received the combined modality therapy. These results demonstrated that combined RT and local IL-12 treatment enhanced the Th1 immune response in immunosuppressive TRAMP-C1 tumors. However, IL-12 alone and the combined modality therapy upregulated the immune checkpoint molecules in tumors (Figure 6). This indicated that IL-12 also led to T cell exhaustion simultaneously with tumor regression. Yang et al. demonstrated that, in patients with follicular B cell non-Hodgkin lymphoma, prolonged exposure to IL-12 caused T cell dysfunction and induced high expression of TIM-3 on T cells [40]. TIM-3 is an established negative regulator, preventing the overactivation of the immune response after infection with a pathogen. Therefore, overexpression of TIM-3 impeded the anti-tumor activity of the host immune response and led to tumor relapse. Furthermore, when the tumor size reached the nadir at day 20 after combined modality therapy, the number of CD8+ T cells in the tumors declined to a level similar to that observed in control tumors. The decrease in CD8+ T cells may be due to the high expression of PD-L1 in tumors with combined modality therapy at day 7 post treatment (Figure 6), which triggered the subsequent T cell apoptosis. These effects may be responsible for tumor recurrence in mice that received the combined modality therapy, though tumors showed a strong response to combined treatment in the early period post treatment.

Prostate tumors show a poor response to immunotherapy compared with other cancers due to their immunosuppressive microenvironment [4,5,6]. Therefore, the combination approaches were investigated to improve the therapeutic efficacy on prostate cancer. Intratumoral injection of adenoviral interleukin-3 provided a more significant tumor growth delay than radiation alone [41]. Hypofractionated RT combined with anti-PD-L1 immunotherapy did not further enhance the tumor growth delay compared with monotherapy in TRAMP-C1 tumor model [42]. In castration-resistant prostate cancer model, immunocheckpoint inhibition PD-1 and PD-L1 combined with RT significantly increased median overall survival compared to immunocheckpoint inhibition therapy alone, and induced the abscopal effect on unirradiated tumors [43]. The present study demonstrated that the combination of IL-12 with RT resulted in a significant reduction of tumor size by inducing Th1 immune response. These indicated that the strategy of radiation combined with immunotherapy has a great potential to enhance the treatment efficacy on local prostate tumors and even metastatic tumors.

In summary, this study demonstrated that local IL-12 immunotherapy enhanced the killing of radio-resistant prostate tumors, increased the coverage of vessels by α-SMA+ pericytes, and enhanced the vascular perfusion. This evidence provides a therapeutic window for additional treatment to improve tumor control. Furthermore, the major advantage of the combination of IL-12 with RT is the reduction of IL-12-associated toxicity to normal tissues, which will benefit the clinical application of this treatment.

## 4. Materials and Methods

### 4.1. Animals

Eight-week-old male C57BL/6J mice were purchased from the National Laboratory Animal Center, Taiwan. All animal experiments were performed according to the animal experimental guidelines set by and with the approval of the Institutional Animal Care and Use Committee of Chang Gung University, Taiwan (IACUC: CGU14-180).

### 4.2. Cell Line Culture

TRAMP-C1 cells (CRL-2730, ATCC, Manassas, VA, USA) were maintained in Dulbecco’s modified eagle’s medium (DMEM) (Gibco, Waltham, MA, USA) containing 10% FBS (Gibco), 1% penicillin-streptomycin (Gibco), 5 μg/mL insulin (Sigma, St. Louis, MO, USA), and 10^−8^ M dehydrotestosterone (Sigma) and incubated at 37 °C in humidified 5% CO_2_/air atmosphere.

### 4.3. Tumor Inoculation and Treatment

**Figure 7:**
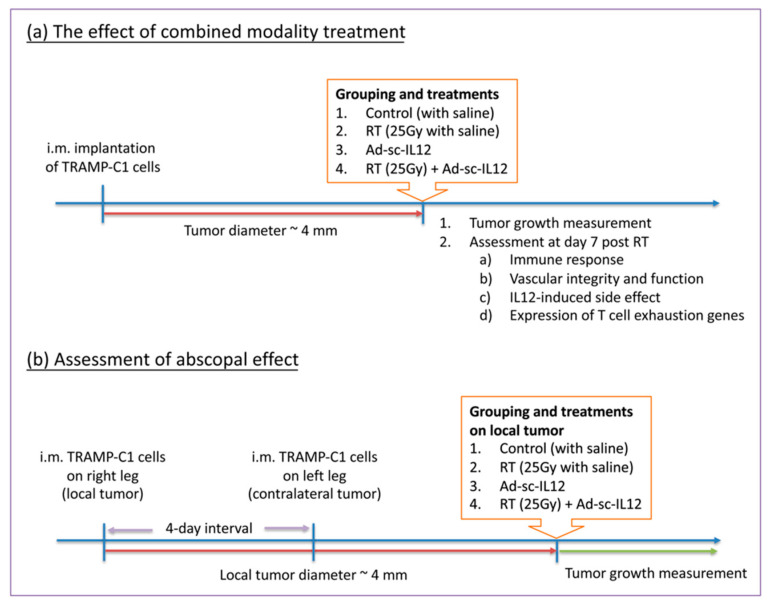
The flow chart for animal experiments in (**a**) the effect of combined modality treatment and (**b**) the assessment of abscopal effect.

To examine the effect of combined modality treatment, TRAMP-C1 cells (3×10^6^ cells in 0.1 mL PBS) were implanted intramuscularly (i.m.) in the right leg of C57BL/6J mice. All of the treatments were initiated when tumor diameter reached 4 mm in average. Mice were randomized into four groups, including control, radiation therapy (a single, high dose of 25 Gy or fractionated dose of 8.9 Gy per fraction for five times), IL-12 treatment, and combined modality with radiation therapy and IL-12 treatment. The protocol of irradiation was described clearly in our previous publication [30]. Briefly, tumor-bearing mice were anesthetized by a mixture (1:1) of ketamine (50 mg/kg, Merial Laboratoire de Toulouse, Toulouse, France) and xylazine (20 mg/kg, Bayer HealthCare Animal Health, Leverkusen, NRW, Germany) and were restrained during irradiation. The tumors were covered with a 1-cm bolus on the surface and irradiated by 6 MV X-rays from a linear accelerator with a dose rate of 2–3 Gy/min. Ad-sc-IL12 adenovirus was kindly provided by Prof. Min-Hua Tao, Academia Sinica, Taipei, Taiwan. For IL-12 treatment, normal saline or Ad-sc-IL12 virus (1 × 10^8^ pfu per mouse) was intratumorally injected at three different parts of tumors, post irradiation. Tumor growth was determined by measuring diameters in two dimensions with a vernier caliper, with at least three mice for each time point. For assessment of abscopal effect, mice were first inoculated with TRAMP-C1 cells (3 × 10^6^ cells in 0.1 mL PBS) in their right legs (local site) and received treatments when the local tumor size reached 4 mm. Four days after injection of tumor cells in the right legs, an equal number of TRAMP-C1 cells were inoculated into the left legs (contralateral site) and tumor growth was monitored. The experimental flowchart is shown in Figure 7. 

### 4.4. Flow Cytometry

Tumors were digested into single-cell suspension by dispase buffer (Gibco), filtered through 100-μm cell strainer (BD Falcon, Franklin Lakes, NJ, USA) to remove the debris, and incubated with RBC lysis buffer (eBioscience, Carlsbad, CA, USA) to remove red blood cells. To avoid non-specific binding, cells were incubated in PBS containing anti-mouse CD16/CD32 antibody (BD Pharmingen, San Jose, CA, USA) and goat serum (Gibco) on ice for 30 min. Cell suspensions were stained with APC-CD8 antibody (BD Pharmingen) on ice for 1 hr, and then washed by PBS twice. FACS analysis was performed on BD LSRFortessa flow cytometer (Becton Dickinson, Franklin Lakes, NJ, USA) and data were analyzed by FACSDiva software (Becton Dickinson).

### 4.5. IL-12 and IFN-γ Measurement

Whole blood was collected and centrifuged at 3000 rpm for 5 min to isolate the serum and the serum was stored at −80 °C. The amount of IL-12 and IFN-γ was measured by mouse IL-12 and IFN-γ ELISA assay (R&D system, Minneapolis, MN, USA) according to the manufacturer’s protocol.

### 4.6. Immunohistochemical Analysis

Mice were injected with intraperitoneally pimonidazole hydrochloride (PIMO, 2 mg per mouse, HypoxyprobeTM-1 Kit, Hypoxyprobe, Inc, Burlington, MA, USA) and intravenously with Hoechst33342 (0.375 mg per mouse, Thermo Fisher, Waltham, MA, USA) 1 h and 1 min before sacrifice, respectively, to label the hypoxia and blood perfusion region. Tumor tissues embedded in OCT compound (Sakura Finetek, Torrance, CA, USA) were cut into 10-μm-thick sections. Frozen sections were fixed in cold 100% methanol for 5 min and washed by PBS. To avoid non-specific binding, tissue slides were mounted with PBS containing 1% BSA (Sigma) and 0.01% Tween-20 (Sigma) for 60 min at room temperature. The slides were subsequently incubated at 4 °C overnight with specific first antibody against CD8, CD31 (BD Pharmingen), α-SMA (Sigma), and pimonidazole (HypoxyprobeTM-1 Kit, Hypoxyprobe, Inc.), and primary antibodies were detected by specific secondary antibodies conjugated with Alexa Fluor 594 or Alexa Fluor 488 (Invitrogen, Waltham, MA, USA) for 30 min at room temperature. After washing by PBS and air drying, the slides were mounted with mounting medium containing DAPI (Invitrogen) for nuclei stain or without DAPI for Hoechst33342 dye perfusion imaging. Images were captured by fluorescence microscope (Nikon, Tokyo, Japan) and ImageXpress Micro Confocal system (Molecular Devices, San Jose, CA, USA), and analyzed by Image-Pro Plus 6.0 software (Media Cybernetics, Inc., Rockville, MD, USA).

### 4.7. Liver Damage Analysis

The blood collected in Li-heparin-containing blood collection tube (AmiShield, Taoyuan, Taiwan) was centrifuged at 1500 rpm for 10 min to isolate the plasma. The level of ALT in plasma was determined by a biochemical analyzer using Liver Shield panel kit (AmiShield) according to the manufacturer’s protocol.

### 4.8. RNA Isolation and Real-Time PCR

Mice were sacrificed at day 7 post irradiation and total RNA of the tumors isolated by TRIzol (Invitrogen) was reverse transcripted into cDNA by Omniscript reverse transcriptase kit (Qiagen, Düsseldorf, NRW, Germany). The quantitative PCR performed by the LightCycler^®^ 480 SYBR Green I Master reagent (Roche, Basel, Switzerland) was analyzed by CFX Connect™ Real-Time PCR Detection System (Bio-Rad, Hercules, CA, USA). The mRNA expression of each gene was normalized for the housekeeping gene (β-actin) and the fold change of gene expression in each group was determined by the difference (ΔΔCt value) compared to the control group. The primers of genes are shown in Table 1.

### 4.9. Statistics

The statistical significance of differences was determined by one-way ANOVA using GraphPad Prism 8 (GraphPad, San Diego, CA, USA). All results are presented as mean ± standard error of the mean. Differences were considered statistically significant when *p*-values ≤ 0.05.

## Figures and Tables

**Table 1:** Primer sequences of real-time PCR.

Gene Name	Sequence
β-actin forward	ACCCTAAGGCCAACCGTGAA
β-actin reverse	ATGGCGTGAGGGAGAGCATAG
PD-L1 forward	GCTCCAAAGGACTTGTACGTG
PD-L1 reverse	TGATCTGAAGGGCAGCATTTC
PD-1 forward	TGATCTGAAGGGCAGCATTTC
PD-1 reverse	CATTTGCTCCCTCTGACACTG
TIM-3 forward	CCACGGAGAGAAATGGTTC
TIM-3 reverse	CATCAGCCCATGTGGAAAT
LAG-3 forward	CTGGGACTGCTTTGGGAAG
LAG-3 reverse	GGTTGATGTTGCCAGATAACCC

## Data Availability

The data presented in this study are available within the article text, figures, and Supplementary Materials.

## Supplementary Materials

The following are available online at https://www.mdpi.com/article/10.3390/ijms221810053/s1. Figure S1. IL-12 treatment delayed the decline of microvascular density post irradiation. Figure S2. The expression of MIG and IP-10 was up-regulated in tumors with IL-12 treatment.
