# Supplementary material for: Local Interleukin-12 Treatment Enhances the Efficacy of Radiation Therapy by Overcoming Radiation-Induced Immune Suppression
Source: Int J Mol Sci. 2021 Sep 17;22(18):10053. doi: 10.3390/ijms221810053 (PMC8468040; doi:10.3390/ijms221810053)
Supplement: Supplementary file 1 [file ijms-22-10053-s001.zip › Supplementary materials.pdf]

Supplementary Materials

Figure S1. IL-12 treatment delayed the decline of microvascular density post irradiation. MVD of tumors was examined at day 1, day 7 and day 10 post-treatment by staining with endothelial cell marker, CD31. Relative MVD at indicated point was defined as the ratio of MVD of RT, IL-12 and combined modality compared to control group. N≥ 3 in each group. *P <0.05, **P <0.01.

Figure S2. The expression of MIG and IP-10 was up-regulated in tumors with IL-12 treatment. Gene expression of MIG and IP-10 in tumors of each group was examined by real-time PCR at day 7 after irradiation. **P <0.01, ***P <0.001. N= 4 in each group.
