# Supplementary figures and images for: Local Interleukin-12 Treatment Enhances the Efficacy of Radiation Therapy by Overcoming Radiation-Induced Immune Suppression
Source: Int J Mol Sci. 2021 Sep 17;22(18):10053. doi: 10.3390/ijms221810053 (PMC8468040; doi:10.3390/ijms221810053)

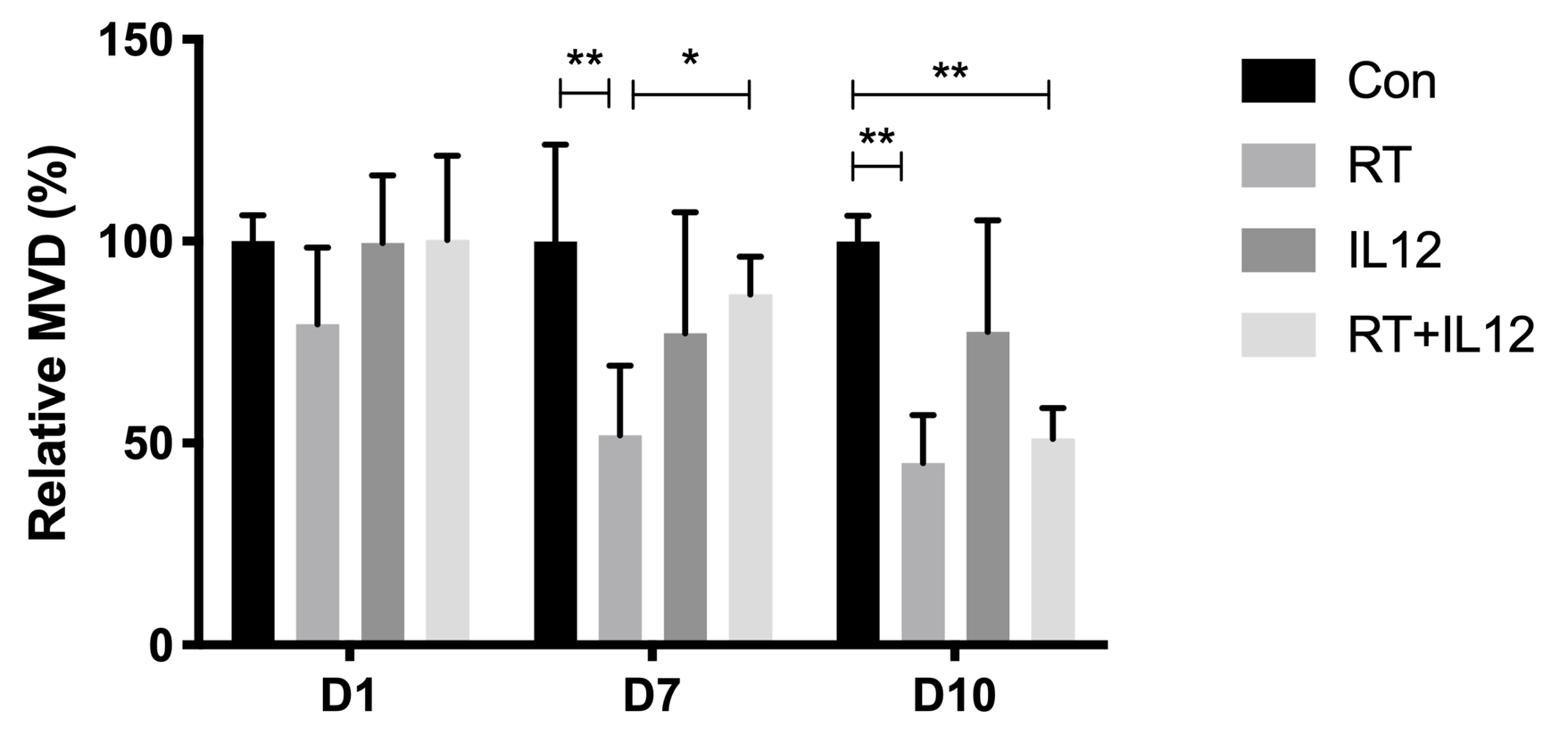

Supplement: Supplementary file 1 [file ijms-22-10053-s001.zip › Fig S1.tif]

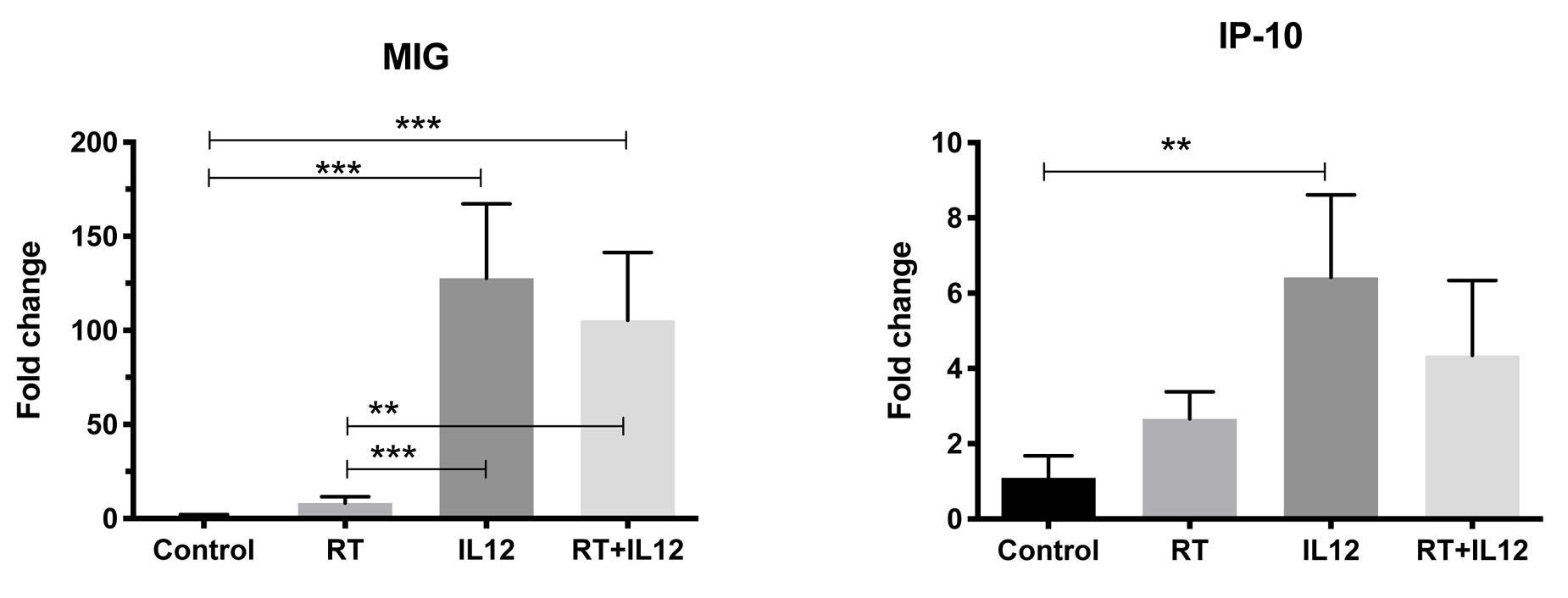

Supplement: Supplementary file 1 [file ijms-22-10053-s001.zip › Fig S2.tif]
